# The effect of aclidinium bromide on daily respiratory symptoms of COPD, measured using the Evaluating Respiratory Symptoms in COPD (E-RS: COPD) diary: pooled analysis of two 6-month Phase III studies

**DOI:** 10.1186/s12931-016-0372-1

**Published:** 2016-05-23

**Authors:** Paul W. Jones, Nancy K. Leidy, Asha Hareendran, Rosa Lamarca, Ferran Chuecos, Esther Garcia Gil

**Affiliations:** St George’s, University of London, London, UK; Evidera, Bethesda, MD USA; AstraZeneca PLC, Barcelona, Spain

**Keywords:** Cohort, Retrospective, Prospective, Exacerbation risk, Nighttime symptoms, Morning symptoms

## Abstract

**Background:**

Reducing the severity of respiratory symptoms is a key goal in the treatment of chronic obstructive pulmonary disease (COPD). We evaluated the effect of aclidinium bromide 400 μg twice daily (BID) on respiratory symptoms, assessed using the Evaluating Respiratory Symptoms in COPD (E-RS^™^: COPD) scale (formerly EXACT-RS).

**Methods:**

Data were pooled from the aclidinium 400 μg BID and placebo arms of two 24-week, double-blind, randomized Phase III studies evaluating aclidinium monotherapy (ATTAIN) or combination therapy (AUGMENT COPD I) in patients with moderate to severe airflow obstruction. Patients were stratified by Global initiative for chronic Obstructive Lung Disease (GOLD) Groups A–D. Change from baseline in E-RS scores, proportion of responders (patients achieving pre-defined improvements in E-RS scores), and net benefit (patients who improved minus patients who worsened) were analyzed.

**Results:**

Of 1210 patients, 1167 had data available for GOLD classification. Mean (standard deviation) age was 63.2 (8.6) years, 60.7 % were male, and mean post-bronchodilator forced expiratory volume in 1 s was 54.4 % predicted. Compared with placebo, aclidinium 400 μg BID significantly improved RS-Total (2.38 units vs 0.79 units, *p* < 0.001) and domain scores (all *p* < 0.001) at Week 24, and doubled the likelihood of being an RS-Total score responder (*p* < 0.05), irrespective of GOLD group. The net benefit for RS-Total (Overall: 56.9 % vs 19.4 %; A + C: 65.7 % vs 6.3 %; B + D: 56.0 % vs 20.8 %, for aclidinium 400 μg BID and placebo respectively; all *p* < 0.05) and domain scores (all *p* < 0.05) was significantly greater with aclidinium compared with placebo, in both GOLD Groups A + C and B + D.

**Conclusions:**

Aclidinium 400 μg BID significantly improved respiratory symptoms regardless of the patients’ level of symptoms at baseline. Net treatment benefit was similar in patients with low or high levels of symptoms.

**Trial registration:**

ATTAIN (ClinicalTrials.gov identifier: NCT01001494) and AUGMENT COPD I (ClinicalTrials.gov identifier: NCT01437397).

**Electronic supplementary material:**

The online version of this article (doi:10.1186/s12931-016-0372-1) contains supplementary material, which is available to authorized users.

## Background

Respiratory symptoms, including breathlessness, chronic cough, and sputum production, are characteristic features of chronic obstructive pulmonary disease (COPD) [1]. These are generally progressive and become increasingly debilitating as the disease worsens. The presence of respiratory symptoms is associated with poor health outcomes, including reduced health status and an increased exacerbation risk [2–4]. Although the primary outcomes in clinical trials of bronchodilators are typically post-bronchodilator forced expiratory volume in 1 s (FEV_1_) endpoints, symptomatic outcomes may better reflect the impact of treatment on patients’ daily lives. It is therefore important to have validated, reliable tools to assess the effect of treatment on symptoms in clinical trials.

Whilst other patient-reported outcomes, such as the St George’s Respiratory Questionnaire (SGRQ; a measure of health status) [5] and the transition dyspnea index (TDI; a measure of the impact of breathlessness on daily activities) [6], are commonly assessed in COPD clinical trials, until recently there has been no standardized method for quantifying daily respiratory symptoms. The EXAcerbations of Chronic obstructive pulmonary disease Tool (EXACT) is a daily diary which is completed by the patient in the evening, with a recall period of ‘today’ to assess acute exacerbations of COPD and chronic bronchitis [7–9]. Recently, the Evaluating Respiratory Symptoms in COPD (E-RS™^1^: COPD) tool, a derivative of the EXACT and previously referred to as EXACT-RS, was developed to meet the need for a standardized respiratory symptom diary. The E-RS uses the 11 respiratory symptom items from the 14-item EXACT and assesses both overall daily respiratory COPD symptoms (RS-Total score) and specific respiratory symptoms using three subscales (RS-Breathlessness, RS-Cough & Sputum and RS-Chest Symptoms) [10, 11].

Aclidinium bromide 400 μg twice daily (BID) is a long-acting muscarinic antagonist approved as a maintenance bronchodilator treatment in patients with COPD. In this *post-hoc* analysis, we pool data from the aclidinium 400 μg and placebo arms of two 24-week, double-blind, randomized Phase III studies of aclidinium monotherapy (ATTAIN) or combination therapy (AUGMENT COPD I) [12, 13] to evaluate the effect of aclidinium on respiratory symptoms, assessed using the E-RS. Patients were stratified according to Global initiative for chronic Obstructive Lung Disease (GOLD) Groups A–D in order to investigate how RS-Total scores and the effect of aclidinium relate to these patient groups.

Additionally, we investigated the relationship between E-RS scores and other clinical measures at baseline and over time.

## Methods

### Study design

ATTAIN (ClinicalTrials.gov identifier: NCT01001494) and AUGMENT COPD I (ClinicalTrials.gov identifier: NCT01437397) were multi-national, randomized, double-blind, placebo-controlled Phase III studies evaluating aclidinium monotherapy (ATTAIN) [12] or combination therapy (AUGMENT COPD I) [13]. In both studies, following screening and a 2–3-week run-in period, patients were randomized to 24 weeks’ treatment with: aclidinium 200 μg BID, aclidinium 400 μg BID (metered dose; equivalent to aclidinium 322 μg delivered dose) or placebo BID in ATTAIN (1:1:1); and aclidinium bromide/formoterol fumarate (AB/FF) 400/12 μg BID, AB/FF 400/6 μg BID, aclidinium 400 μg BID, formoterol 12 μg BID or placebo BID (1:1:1:1:1) in AUGMENT COPD I. The ACLIFORM COPD study (ClinicalTrials.gov identifier: NCT01462942), which was of similar design to AUGMENT COPD I, was not included in this pooled analysis due to a large and unexplained placebo effect in health status assessments [14]; given the significant correlation between health status and the E-RS [11], it is not clear how the placebo effect impacted E-RS assessments in this study, therefore a decision was made prospectively not to include these data in this analysis.

All treatments were administered via the Genuair^TM^/Pressair®^2^ inhaler. Inhaled albuterol/salbutamol (108/100 μg/puff) was permitted as relief medication, as long as it was discontinued 6 h prior to study visits in both studies. Details of other permitted concomitant and restricted medications have been reported elsewhere [12, 13].

Both studies were conducted in accordance with the Declaration of Helsinki, International Conference on Harmonization/Good Clinical Practice Guidelines and local regulations. The study protocols were approved by Institutional Review Boards/Independent Ethics Committees as required by each country and all patients gave written informed consent.

### Study populations

Detailed inclusion/exclusion criteria for each study have been reported previously [12, 13]. Briefly, both studies enrolled male and female patients (≥40 years old) with a diagnosis of stable COPD and moderate to severe airflow obstruction (FEV_1_ ≥30 % and <80 % of the predicted value and FEV_1_/forced vital capacity ratio <70 %) [1] who were current or former smokers with a smoking history of ≥10 pack-years. A history of respiratory symptoms was not a specific inclusion criterion in either of the studies.

Exclusion criteria included any respiratory tract infection or COPD exacerbation within 6 weeks prior to screening (3 months if the exacerbation resulted in hospitalization), any clinically relevant respiratory or cardiovascular conditions, including a history or current diagnosis of asthma and a history of hypersensitivity to inhaled anticholinergic agents or other inhaled medications.

### Assessments

#### Daily COPD respiratory symptoms

Every evening, patients completed the EXACT diary, from which daily COPD symptom scores were derived using the E-RS scoring algorithms [10, 11]. The RS-Total score is the sum of 11 items that relate specifically to respiratory symptoms (score range, 0–40), with higher scores indicating more severe symptoms. The RS-Breathlessness domain is the sum of five items related to breathlessness (score range, 0–17); the RS-Cough & Sputum domain score is the sum of three items that relate to cough and sputum symptoms (score range, 0–11); and the RS-Chest Symptoms domain score is the sum of three items related to chest congestion/discomfort (score range, 0–12). RS-Total and domain scores were assessed at baseline and over the 24-week study duration. Responder definitions for the E-RS have been proposed and are shown in Table 1. These responder definitions for symptomatic improvement were based on results from three randomized controlled trials in which responder definitions were defined using criterion- and distribution-based methods [11]. For this analysis, responder status was assessed using data at baseline (averaged over the week before randomization) and at the end of the study (averaged over the last week of the study).

**Table 1:** Clinical outcome measure responder definitions

Outcome	Responder definition^b^	Reference
Daily respiratory symptoms^a^		
RS-Total score	≥2-unit decrease from baseline	[11]
RS-Breathlessness domain score	≥1-unit decrease from baseline	
RS-Cough & Sputum domain score	≥0.7-unit decrease from baseline	
RS-Chest Symptoms domain score	≥0.7-unit decrease from baseline	
Health status		
SGRQ total score	≥4-unit decrease from baseline	[19]
Dyspnea		
TDI focal score	≥1-unit increase from baseline	[20]
Lung function		
Trough FEV_1_ (spirometry)	≥100 mL increase from baseline	[21]

^a^Higher scores indicate more severe symptoms

^b^Patients with a *decrease* from baseline that exceeded the pre-defined value were considered to have an improvement (responders). Patients with an *increase* from baseline that exceeded the pre-defined value were considered to have a worsening. Patients with a change from baseline in *either direction* that did not reach the pre-defined value were considered to have no change

*FEV*_*1*_, forced expiratory volume in 1 s; *SGRQ*, St George’s Respiratory Questionnaire; *TDI*, transition dyspnea index

#### Other clinical outcomes

FEV_1_ was measured before the morning dose on Day 1 (baseline) and at each study visit (trough FEV_1_). Dyspnea was assessed at baseline using the baseline dyspnea index (BDI) and changes in dyspnea were assessed with the TDI [6]. Health status was assessed using the SGRQ [5]. Responders for each outcome were defined as patients who achieved a clinically meaningful improvement from baseline (Table 1).

### Endpoints

The endpoints examined in this predefined analysis of data from the ATTAIN and AUGMENT COPD I trials [12, 13], were changes from baseline in RS-Total and domain scores at Week 24 and the percentage of RS-Total and domain score responders at Week 24. In addition, a net treatment benefit was calculated as the proportion of patients who had an improvement minus the proportion of patients who had a worsening. Response and worsening were determined using published threshold estimates for meaningful change (Table 1). Patients with a change from baseline in either direction that did not exceed the responder threshold were considered to have ‘no change’.

The relationship between E-RS responder status at Week 24 and responder status for other clinical outcomes was also evaluated. In addition, baseline E-RS scores were correlated with baseline measures of health status (SGRQ total score), dyspnea (BDI focal score), relief-medication use and airflow obstruction (post-bronchodilator FEV_1_).

Safety and tolerability outcomes have been reported for each of the studies previously [12, 13].

### Statistical analysis

These *post-hoc* analyses were conducted using data from patients randomized to placebo or aclidinium 400 μg BID (the dose licensed for use in patients with COPD). Data from patients in the intent-to-treat (ITT) population (all randomized patients who took at least one dose of double-blind treatment and had a baseline and at least one post-baseline FEV_1_ assessment) who also had baseline SGRQ and E-RS scores were evaluated. All other treatment arms and time points were excluded from analysis. The primary analysis was performed on patients randomized to treatment, but a secondary analysis tested the effect of treatment versus placebo in patients stratified into GOLD Groups A–D based on airflow obstruction, exacerbation risk, and SGRQ total score. GOLD recommends use of the COPD Assessment Test (CAT) score, or the modified Medical Research Council (mMRC) dyspnea grade, but as neither CAT score nor mMRC grades were assessed in ATTAIN or AUGMENT, the SGRQ total score was used as a surrogate to stratify patients [15]. An SGRQ total score of ≥25 was used, as this corresponds with a CAT score of ≥10 [15]. For efficacy analyses, low symptom patients in GOLD Groups A and C were pooled (A + C); similarly, the higher-symptom patients in Groups B and D were pooled (B + D).

Baseline data are reported as mean (standard deviation [SD]) or percentage. Variation in baseline E-RS scores between individual (A, B, C, D) and pooled (A + C and B + D) GOLD groups was analyzed using analysis of covariance (ANCOVA) models, with E-RS score as the dependent variable and GOLD group, study, sex, and tobacco use as factors and age as a covariate. A minimum of four of seven days of diary data were required for computation of baseline scores in both trials.

Changes from baseline in RS-Total and domain scores at Week 24 were analyzed using a mixed model for repeated measures, adjusted for baseline, treatment, visit, sex, age, smoking status, and treatment-by-visit interaction. These are reported as least squares (LS) means (standard error). The proportion of RS-Total and domain, SGRQ, TDI, and trough FEV_1_ responders was analyzed using a logistic random-effects model and data are reported as odds ratio (OR; 95 % confidence intervals).

The relationship between RS-Total and domain score responders and trough FEV_1_, SGRQ, or TDI responders was assessed in the total patient population using a chi-squared test. Pearson’s correlation co-efficient (r) was used to assess the correlation between baseline RS-Total score and SGRQ total score, BDI, relief-medication use, and % predicted post-bronchodilator FEV_1_ at baseline.

## Results

### Patient population

Patient demographic and baseline clinical characteristics for ATTAIN and AUGMENT COPD I are shown in Table 2. Overall, there were 1210 patients in the pooled ITT population. Of these, 1161 patients had baseline E-RS and SGRQ data available and were included in these *post-hoc* analyses (Additional file 1: Table S1). Using baseline SGRQ data, patients were categorized into GOLD groups as follows: GOLD Group A, *n* = 96 patients; GOLD Group B, *n* = 568; GOLD Group C, *n* = 42; and GOLD Group D, *n* = 461. Overall, the mean (SD) age was 63.2 (8.6) years, 60.6 % of patients were male, and mean (SD) post-bronchodilator FEV_1_ was 1.6 (0.5) L.

**Table 2:** Baseline demographic and clinical characteristics by treatment, study and overall

	ATTAIN		AUGMENT COPD		Pooled analysis sample		
Characteristic	Aclidinium 400 μg (*n* = 269)^a^	Placebo (*n* = 273)^a^	Aclidinium 400 μg (*n* = 337)^a^	Placebo (*n* = 331)^a^	Aclidinium 400 μg (*n* = 583)^b^	Placebo (*n* = 578)^b^	All patients (*n* = 1161)^b^
Age, years, mean (SD)	62.9 (8.4)	62.0 (8.0)	64.4 (8.7)	63.5 (8.9)	63.7 (8.6)	62.8 (8.5)	63.2 (8.6)
Male, n (%)	182 (67.7)	189 (69.2)	188 (55.8)	175 (52.9)	356 (61.1)	347 (60.0)	703 (60.6)
Caucasian, n (%)	257 (95.5)	260 (95.2)	314 (93.2)	316 (95.5)	548 (94.0)	552 (95.5)	1100 (94.7)
BMI, kg/m^2^, mean (SD)	27.0 (4.8)	26.6 (5.2)	27.5 (5.3)	27.7 (5.6)	27.3 (5.1)	27.2 (5.5)	27.2 (5.3)
Current smoker, n (%)	148 (55.0)	144 (52.8)	171 (50.7)	169 (51.1)	303 (52.0)	300 (51.9)	603 (51.9)
Smoking history, pack-years, mean (SD)	41.7 (21.1)	38.9 (18.3)	52.0 (26.1)	53.4 (28.5)	47.1 (23.4)	47.1 (25.8)	47.1 (24.6)
Post-bronchodilator FEV_1_, L, mean (SD)^c^	1.6 (0.5)	1.6 (0.5)	1.5 (0.5)	1.6 (0.5)	1.6 (0.5)	1.6 (0.5)	1.6 (0.5)
Post-bronchodilator FEV_1_, % predicted, mean (SD)^c^	56.2 (12.2)	56.6 (12.8)	53.0 (13.3)	52.6 (13.3)	54.5 (12.9)	54.2 (13.3)	54.4 (13.1)
% bronchial reversibility (SD)	11.3 (12.9)	12.3 (15.7)	19.1 (16.5)	18.4 (15.2)	15.7 (15.6)	15.7 (15.7)	15.7 (15.6)
Number of exacerbations in previous year, mean (SD)	0.5 (0.7)	0.4 (0.9)	0.3 (0.8)	0.3 (0.6)	0.4 (0.8)	0.3 (0.7)	0.4 (0.8)

^a^Patients from the ITT population

^b^Patients from the pooled subpopulation: ITT population (*N* = 1210) with data available for GOLD classification (43 patients were excluded due to missing GOLD data and a further 6 patients due to missing baseline E-RS data)

^c^At screening visit

*BMI*, body mass index; *COPD*, chronic obstructive pulmonary disease; *E-RS*, Evaluating Respiratory Symptoms; *FEV*_*1*_, forced expiratory volume in 1 s; *GOLD*, Global initiative for chronic Obstructive Lung Disease; *ITT*, intent-to-treat; *SD* standard deviation

### Baseline E-RS scores

**Fig. 1:**
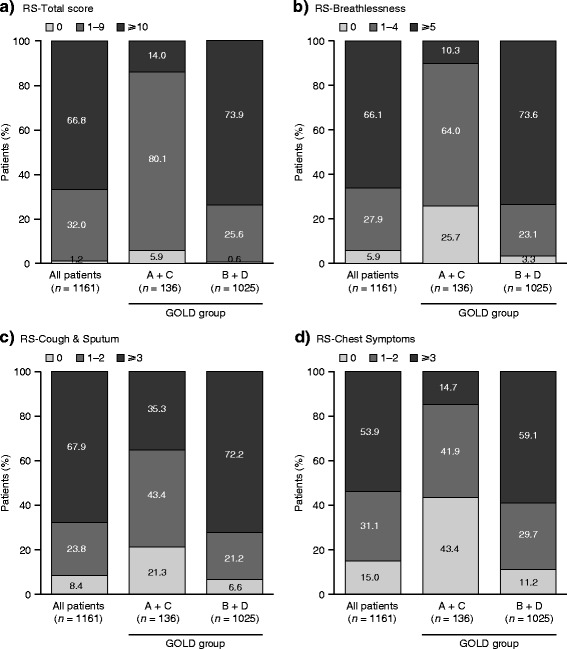
Distribution of E-RS scores at baseline, overall and by pooled GOLD group. *n* = patients with available data. **a** RS Total score, ranged 0 to 40. **b** RS-Breathlessness domain score, range 0 to 17. **c** RS-Cough & Sputum domain score, range 0 to 11. **d** RS-Chest Symptoms domain score, range 0 to 12. Higher scores indicate more severe symptoms. *E-RS*, Evaluating Respiratory Symptoms; *GOLD*, Global initiative for chronic Obstructive Lung Disease

Mean E-RS scores at baseline are shown in Table 3. At baseline, overall mean (SD) RS-Total score was 12.6 ± 6.6 (range 0–34), but there was a significant difference in RS-Total and domain scores between GOLD groups (ANCOVA, *p* < 0.001 for total score and all domains). The RS-Total and domain scores were higher in Groups B and D compared with Groups A and C (all *p* < 0.001). In addition, RS-Total and RS-Breathlessness scores were higher in Group D (higher symptom higher risk) compared with Group B (higher symptom lower risk) (both *p* < 0.001). Baseline RS-Total and domain scores were balanced between treatment arms in each pooled GOLD group (A + C and B + D), with the exception of RS-Cough & Sputum domain scores in patients in GOLD Group A + C, which were significantly greater in the aclidinium group compared with placebo (*p* < 0.05; Additional file 1: Table S2). The distribution of baseline E-RS scores by pooled GOLD group is shown in Fig. 1, and analyzing the data by individual GOLD group yields similar results (Additional file 1: Figure S1).

**Table 3:** Baseline E-RS scores, overall and by GOLD group

	RS-Total^a^	RS-Breathlessness^b^	RS-Cough & Sputum^c^	RS-Chest Symptoms^d^
	mean (SD)	mean (SD)	mean (SD)	mean (SD)
All patients				
Placebo (*n* = 578)	12.4 (6.5)	6.3 (3.6)	3.4 (1.9)	2.7 (2.0)
Aclidinium 400 μg BID (*n* = 583)	12.7 (6.8)	6.4 (3.7)	3.5 (1.9)	2.9 (2.1)
Total (*n* = 1161)	12.6 (6.6)	6.3 (3.6)	3.4 (1.9)	2.8 (2.0)
GOLD Group A				
Placebo (*n* = 52)	4.6 (3.2)	2.0 (1.7)	1.6 (1.3)	0.9 (1.2)
Aclidinium 400 μg BID (*n* = 42)	6.0 (5.2)	2.3 (2.7)	2.3 (1.8)	1.4 (1.7)
Total (*n* = 94)	5.2 (4.3)	2.1 (2.2)	2.0 (1.6)	1.1 (1.5)
GOLD Group B				
Placebo (*n* = 270)	13.1 (5.9)	6.5 (3.2)	3.6 (1.8)	3.0 (1.8)
Aclidinium 400 μg BID (*n* = 296)	13.1 (6.5)	6.4 (3.5)	3.6 (1.9)	3.1 (2.0)
Total (*n* = 566)	13.1 (6.2)	6.4 (3.3)	3.6 (1.8)	3.1 (1.9)
GOLD Group C				
Placebo (*n* = 20)	6.5 (4.4)	3.0 (2.1)	2.0 (1.6)	1.5 (1.5)
Aclidinium 400 μg BID (*n* = 22)	6.0 (3.4)	2.3 (1.7)	2.4 (1.5)	1.3 (1.2)
Total (*n* = 42)	6.2 (3.9)	2.6 (1.9)	2.2 (1.6)	1.4 (1.4)
GOLD Group D				
Placebo (*n* = 236)	13.9 (6.4)	7.3 (3.5)	3.6 (1.8)	2.9 (2.0)
Aclidinium 400 μg BID (*n* = 223)	14.2 (6.5)	7.5 (3.4)	3.6 (2.0)	3.2 (2.1)
Total (*n* = 459)	14.0 (6.5)	7.4 (3.5)	3.6 (1.9)	3.0 (2.1)

*n* = patients with available data

^a^RS-Total score ranged from 0 to 40

^b^RS-Breathlessness domain score ranged from 0 to 17

^c^RS-Cough & Sputum domain score ranged from 0 to 11

^d^RS-Chest Symptoms domain score ranged from 0 to 12

Higher scores indicate more severe symptoms

RS-Total and domain scores were higher in GOLD Groups B and D compared with Groups A and C (all *p* < 0.001; ANCOVA). RS-Total and RS-Breathlessness scores were higher in GOLD Group D compared with Group B (both *p* < 0.001; ANCOVA)

*ANCOVA*, analysis of covariance; *BID*, twice daily; *E-RS*, Evaluating Respiratory Symptoms; *GOLD*, Global initiative for chronic Obstructive Lung Disease; *SD*, standard deviation

### Efficacy analyses

**Fig. 2:**
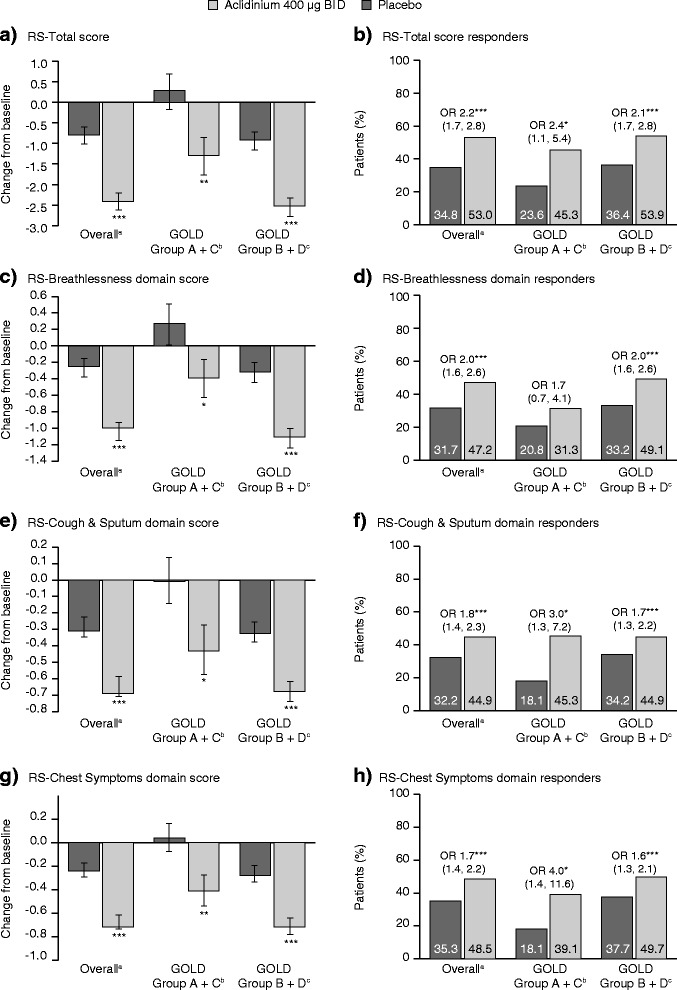
Change from baseline in E-RS scores and proportion of E-RS responders at Week 24, overall and by GOLD group **a** and **b** RS-Total score; **c** and **d** RS-Breathlessness domain; **e** and **f** RS-Cough & Sputum domain; **g** and **h** RS-Chest Symptoms domain. *p < 0.05, **p < 0.01, ***p < 0.001 vs placebo. Change from baseline data are LS means (SE). Responder data are OR (95 % CI). ^a^placebo *n* = 578; aclidinium *n* = 583; ^b^placebo *n* = 72; aclidinium *n* = 64; ^c^placebo *n* = 506; aclidinium *n* = 519. BID, twice daily; *CI*, confidence interval; *E-RS*, Evaluating Respiratory Symptoms; *GOLD*, Global initiative for chronic Obstructive Lung Disease; *LS*, least squares; *OR*, odds ratio; *SE*, standard error

Changes from baseline in RS-Total and domain scores at Week 24 are shown in Fig. 2. After 24 weeks, in the overall patient population, the improvement from baseline in RS-Total score was significantly greater with aclidinium compared with placebo (*p* < 0.001; Fig. 2a); 2.38 units with aclidinium versus 0.79 units with placebo. For each of the E-RS domains, improvements from baseline in E-RS score in the overall population were also significantly greater with aclidinium (all *p* < 0.001; Fig. 2c, e, g). The magnitude of the treatment differences with aclidinium versus placebo for RS-Total score and each of the E-RS domains were numerically similar, irrespective of GOLD group (Fig. 2a, c, e, g).

Overall, patients receiving aclidinium were significantly more likely to be an RS-Total score responder compared with patients receiving placebo (OR 2.2, *p* < 0.001; Fig. 2b). Similarly, patients in the aclidinium group were approximately twice as likely to be responders in the three domain scores compared with placebo (OR 1.7–2.0, all *p* < 0.001; Fig. 2d, f, h).

When assessed according to GOLD group, with the exception of the RS-Breathlessness domain score in patients in GOLD Group A + C (OR 1.6, *p* > 0.05; Fig. 2d), the likelihood of achieving the pre-defined improvement from baseline in RS-Total and domain scores was also significantly greater with aclidinium compared with placebo in GOLD Group A + C and GOLD Group B + D (OR 1.7–3.6, all *p* < 0.05; Fig. 2b, d, f, h).

**Fig. 3:**
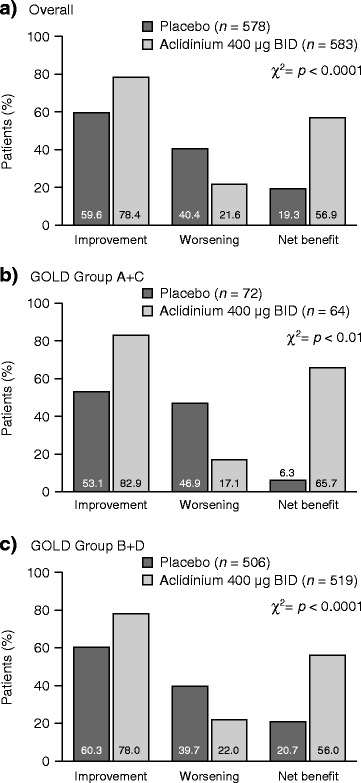
Patients with improvement and worsening in RS-Total score, and net treatment benefit at Week 24, overall (**a**) and by GOLD group (**b** and **c**). BID, twice daily; *GOLD*, Global initiative for chronic Obstructive Lung Disease

**Fig. 4:**
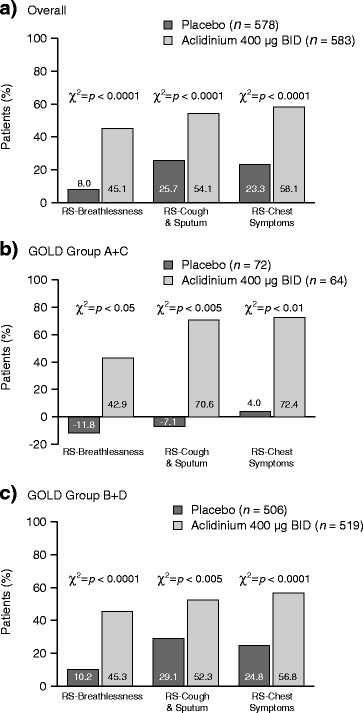
Net treatment benefit for E-RS domain scores at Week 24, overall (**a**) and by GOLD group (**b** and **c**) *n* = patients with available data. BID, twice daily; *E-RS*, Evaluating Respiratory Symptoms; *GOLD*, Global initiative for chronic Obstructive Lung Disease

The net benefit in RS-Total score was significantly higher in patients treated with aclidinium than in those receiving placebo. This was seen in the whole treatment population and in patients classified as GOLD Group A + C and B + D (all *p* < 0.01; Fig. 3a–c). A similar pattern was observed for the E-RS domains (all *p* < 0.05; Fig. 4a–c).

### Association between E-RS responder status and other clinical outcomes

There were significant associations between RS-Total score responder status at Week 24 and responder status for SGRQ total score, TDI focal score and trough FEV_1_ (all *p* ≤ 0.002; Additional file 1: Figure S2a). The strongest association was observed between responder status for RS-Total score and SGRQ total score (*χ*^2^ = 118.9, *p* < 0.001).

There was also a significant relationship between responder status for each E-RS domain and responder status for the SGRQ total and TDI focal scores (all *p* < 0.05; Additional file 1: Figure S2b–d). A significant association was seen between E-RS Breathlessness domain responders and trough FEV_1_ responders (*p* = 0.001; Additional file 1: Figure S2b), but no association was found between RS-Chest Symptoms and RS-Cough & Sputum responders and trough FEV_1_ responders (Additional file 1: Figure S2c–d).

### Correlation between E-RS scores and other clinical outcomes

Baseline RS-Total and domain scores were significantly correlated with baseline SGRQ total score, BDI focal score, relief-medication use and FEV_1_ (all *p* < 0.05; Additional file 1: Table S3).

## Discussion

In this pooled analysis, after 6 months of treatment, aclidinium 400 μg BID significantly improved RS-Total and domain scores compared with placebo and increased the proportion of patients who achieved pre-defined improvements from baseline in E-RS scores. Pooling data from two studies made available sufficient data for sub-analysis by GOLD group, which demonstrated that aclidinium can improve respiratory symptoms in patients with both low symptoms (GOLD Group A + C) or high symptoms (GOLD Group B + D).

Symptoms are usually assessed in clinical trials by asking patients to recall their symptoms over a specified period (e.g. since the last visit, past month, previous week), but periodic assessment may be subject to recall bias, with patients tending to report more precisely symptoms that they experienced in the most recent past. The E-RS was designed to meet the need for a daily respiratory symptoms diary and, whilst its validity and reliability has been demonstrated [10, 11], this is the first test of its ability to capture treatment effects.

Criteria for defining RS-Total and domain score responders have been proposed [11] and in the overall patient population, and in patients in GOLD Group B + D, improvements from baseline in RS-Total and domain scores with aclidinium met or exceeded the thresholds for defining responders. Aclidinium improved daily respiratory symptoms in all groups irrespective of baseline symptom severity. However, although the largest changes were seen in those with more severe symptoms at baseline, it should be noted that the number of patients in the low symptom group was relatively small. After 6 months, there was also a net benefit of aclidinium on RS-Total score in both Groups A + C and B + D, taking into account both those who responded compared with no change, and no change compared with those who deteriorated. Taken together, these results suggest aclidinium improves daily respiratory symptoms, regardless of symptom severity at baseline, although results for the RS-Cough & Sputum domain for GOLD Group A + C need to be interpreted with caution because of the imbalance between treatment groups at baseline. In GOLD Groups A and C, the domain in which most patients scored highest was Cough & Sputum, suggesting that this may represent the major cause of respiratory symptoms in these patients. However, it should be noted that there may be a bias introduced here by the method of patient attribution to GOLD group. Whilst the SGRQ does have items concerning cough and sputum, more of them are related to breathlessness and activity, therefore people diagnosed with COPD who have low SGRQ scores are likely to have some respiratory symptoms (otherwise they may not have been diagnosed) and a higher level of cough and sputum may be more apparent in that subgroup.

This analysis also provides further data concerning the validity of E-RS scores. A strong association was seen between E-RS Total score responder status and SGRQ total responder status. Most patients with symptomatic improvement also experienced improvements in health status, although further study of E-RS responders who did not experience meaningful improvements in health status and E-RS non-responders who reported improvements in health status is warranted.

These data also offer insight into the symptomatic manifestations of GOLD Groups A–D when health status is the grouping criterion. RS-Total and domain scores were higher in patients with poorer health status (Group B + D), indicating that these patients had more severe respiratory symptoms, including breathlessness, cough and sputum, and chest symptoms, although this does not necessarily mean that all of these patients experienced severe symptoms, or that those with better health status (Group A + C) were symptom free. Approximately 20–30 % of patients in GOLD Group B + D had an RS-Total score ≤10, and 13–17 % of those in Group A + C had an RS-Total score ≥10. Previous studies have shown that, whilst there is a good degree of concordance between patients categorized into GOLD groups based on breathlessness (mMRC score ≥1) or health status (CAT score ≥10), the distribution of patients is not identical [15, 16]. The results reported here are consistent with these studies and also suggest that groupings based on the cardinal symptoms of COPD (i.e. breathlessness, cough and sputum, and chest symptoms) may be more effective than either dyspnea alone or the broader construct of health status. It is also important to note that the E-RS thresholds used here to designate ‘high’ or ‘low’ symptoms were exploratory and were based on historical score distributions, with further study warranted.

Our findings confirm those of an earlier study, which demonstrated that E-RS scores were significantly correlated with other clinical outcomes, including health status, relief-medication use and alternate measures of symptom severity [11]. The strongest correlations were between baseline E-RS scores and SGRQ total score, consistent with several previous studies that have also shown an association between COPD symptoms and health status [16–18]. In contrast, the relationship between FEV_1_ and respiratory symptoms was weak, consistent with previous research, including a recent observational study which demonstrated that, in patients in clinical practice, the presence of respiratory symptoms was similar in patients across all severities of airflow obstruction [18]. Together, these results highlight the importance of broad assessment of patients’ FEV_1_ and symptoms, consistent with the GOLD strategy document [1].

## Conclusions

In this pooled *post-hoc* analysis of two randomized controlled trials, E-RS scores detected statistically significant and meaningful improvements in daily respiratory symptoms with aclidinium 400 μg BID compared with placebo in patients with moderate to severe COPD. This benefit appears to be irrespective of symptom severity at baseline. Total and domain scores also provided interesting insight into the symptomatic manifestations of GOLD Groups A–D. Results support the use of the E-RS: COPD as a measure of respiratory symptoms in clinical trials of COPD.

## Additional file

Additional file 1: Supplementary information. (DOCX 1244 kb)

## Abbreviations

AB/FF: aclidinium bromide/formoterol fumarate
ANCOVA: analysis of covariance
BDI: baseline dyspnea index
BID: twice daily
BMI: body mass index
CAT: COPD assessment test
CI: confidence interval
COPD: chronic obstructive pulmonary disease
E-RS: Evaluating Respiratory Symptoms
EXACT: EXAcerbations of Chronic obstructive pulmonary disease Tool
FEV_1_: forced expiratory volume in 1 second
GOLD: Global initiative for chronic Obstructive Lung Disease
ITT: intent-to-treat
LS: least squares
mMRC: modified Medical Research Council
OR: odds ratio
SD: standard deviation
SE: standard error
SGRQ: St George’s Respiratory Questionnaire
TDI: transition dyspnea index

## Footnotes

1 The EXACT™ and E-RS™ are owned by Evidera. Permission to use these instruments may be obtained from Evidera (exactpro@evidera.com).

2 Registered trademark of AstraZeneca group of companies; for use within the USA as Pressair®; and as Genuair™ within all other licensed territories.
