# Supplementary material for: The effect of aclidinium bromide on daily respiratory symptoms of COPD, measured using the Evaluating Respiratory Symptoms in COPD (E-RS: COPD) diary: pooled analysis of two 6-month Phase III studies
Source: Respir Res. 2016 May 23;17:61. doi: 10.1186/s12931-016-0372-1 (PMC4877996; doi:10.1186/s12931-016-0372-1)

**Additional File 1: Online supplement**

**Results**

**Correlation between E-RS scores and other clinical measures at baseline**

When the correlation between baseline RS-Total score and other baseline parameters was assessed, the strongest correlation in the overall population was with SGRQ total score (r=0.666, p<0.01; Table S3). There was a significant, though weaker, correlation between baseline RS-Total score and BDI score, relief-medication use and % predicted post-bronchodilator FEV_1_ (Table S3; all p<0.01). A similar pattern was observed with baseline E-RS domain scores (Table S3). SGRQ total score, BDI focal score, relief-medication use and % predicted FEV_1_ were all most strongly correlated with the RS-Breathlessness domain score (r=0.682, r=-0.525, r=0.390 and r=-0.221, respectively; all p<0.01; Table S3).

**Table S1.** Baseline demographic and clinical characteristics, overall and by GOLD group

|  |  | **GOLD Group** | | | |
| --- | --- | --- | --- | --- | --- |
| **Characteristic** | **All patients (n=1,161)^a^** | **A (n=94)** | **B (n=566)** | **C (n=42)** | **D (n=459)** |
| Age, years, mean (SD) | 63.2 (8.6) | 64.9 (8.3) | 63.0 (8.7) | 64.6 (7.1) | 63.1 (8.6) |
| Male, n (%) | 703 (60.6) | 62 (66.0) | 334 (59.0) | 25 (59.5) | 282 (61.4) |
| Caucasian, n (%) | 1,100 (94.7) | 91 (96.8) | 540 (95.4) | 41 (97.6) | 428 (93.2) |
| BMI, kg/m^2^, mean (SD) | 27.2 (5.3) | 26.5 (4.7) | 27.8 (5.4) | 26.0 (4.5) | 26.8 (5.2) |
| Current smoker, n (%) | 603 (52.0) | 42 (44.7) | 296 (52.3) | 22 (52.4) | 243 (52.9) |
| Smoking history,  pack-years, mean (SD) | 47.1 (24.6) | 50.0 (26.5) | 45.9 (24.6) | 50.8 (23.8) | 47.7 (24.2) |
| Post-bronchodilator FEV_1_,^b^ L, mean (SD) | 1.6 (0.5) | 1.9 (0.5) | 1.8 (0.5) | 1.3 (0.3) | 1.2 (0.4) |
| Post-bronchodilator FEV_1_ % predicted,^b^ mean (SD) | 54.4 (13.1) | 64.2 (7.8) | 63.1 (7.8) | 43.3 (7.7) | 42.5 (8.5) |
| % Bronchial reversibility | 15.7 (15.6) | 14.1 (11.4) | 12.5 (13.3) | 20.5 (19.4) | 19.5 (17.6) |
| Number of exacerbations in previous year,  mean (SD) | 0.4 (0.8) | 0.1 (0.4) | 0.2 (0.4) | 0.3 (0.7) | 0.6 (1.1) |
| ≥2 COPD exacerbations in previous year, n (%) | 76 (6.5) | 0 (0.0) | 0 (0.0) | 4 (9.5) | 72 (15.7) |

^a^ Patients from the pooled ITT population (N=1,210) with data available for GOLD classification (43 patients were excluded due to missing GOLD data and a further 6 patients due to missing baseline E-RS data).

^b^At screening visit.
BMI, body mass index; COPD, chronic obstructive pulmonary disease; E-RS, Evaluating-Respiratory Symptoms; FEV_1_, forced expiratory volume in 1 second; GOLD, Global initiative for chronic Obstructive Lung Disease; ITT, intent-to-treat; SD, standard deviation.

**Table S2.** Baseline E-RS scores, overall and by GOLD classification

|  | All patients | | | | | | GOLD Group A+C | | | | GOLD Group B+D | | | | |
| --- | --- | --- | --- | --- | --- | --- | --- | --- | --- | --- | --- | --- | --- | --- | --- |
| E-RS parameter | **Placebo (n=578)** | | **Aclidinium 400 μg BID (n=583)** | | | **Total (n=1,161)^a^** | **Placebo (n=72)** | **Aclidinium 400 μg BID (n=64)** | | **Total (n=136)** | **Placebo (n=506)** | **Aclidinium 400 μg BID (n=519)** | | | **Total (n=1,025)** |
| RS-Total score | | 12.4 (6.5) | | 12.7 (6.8) | 12.6 (6.6) | | 5.1 (3.7) | 6.0 (4.7) | 5.5 (4.2) | | 13.4 (6.1) | | 13.6 (6.5) | 13.5 (6.3) | |
| RS-Breathlessness domain | | 6.3 (3.6) | | 6.4 (3.7) | 6.3 (3.6) | | 2.3 (1.9) | 2.3 (2.4) | 2.3 (2.1) | | 6.9 (3.4) | | 6.9 (3.5) | 6.9 (3.4) | |
| RS-Cough & Sputum domain | | 3.4 (1.9) | | 3.5 (1.9) | 3.4 (1.9) | | 1.7 (1.4) | 2.4 (1.7) | 2.0 (1.6) | | 3.6 (1.8) | | 3.6 (1.9) | 3.6 (1.9) | |
| RS-Chest Symptoms domain | | 2.7 (2.0) | | 2.9 (2.1) | 2.8 (2.0) | | 1.1 (1.3) | 1.3 (1.6) | 1.2 (1.4) | | 3.0 (1.9) | | 3.1 (2.0) | 3.1 (2.0) | |

n=patients with available data.

^a^Patients from the pooled ITT population (N=1,210) with data available for GOLD classification and baseline E-RS (43 patients were excluded due to missing GOLD data and a further 6 patients due to missing baseline E-RS data).
Data are raw means (SD). Higher scores indicate more severe symptoms. Least squares means E-RS Cough & Sputum domain scores were significantly greater in the aclidinium group compared with placebo in GOLD Group A+C (p<0.05; ANCOVA).
ANCOVA, analysis of covariance; BID, twice daily; E-RS, Evaluating-Respiratory Symptoms; GOLD, Global initiative for chronic Obstructive Lung Disease; ITT, intent-to-treat; SD, standard deviation.

**Table S3.** Correlation between baseline E-RS scores and other baseline parameters in the overall population

|  | **Correlation coefficient (Pearson’s r)** | | | |
| --- | --- | --- | --- | --- |
| **E-RS parameter** | **SGRQ total score** | **BDI focal score** | **Relief-medication use** | **% predicted post-bronchodilator FEV_1_** |
| RS-Total score | 0.67 | -0.43 | 0.39 | -0.15 |
| RS-Breathlessness domain score | 0.68 | -0.53 | 0.39 | -0.22 |
| RS-Cough & Sputum domain score | 0.47 | -0.21 | 0.27 | -0.05 |
| RS-Chest Symptoms domain score | 0.53 | -0.29 | 0.34 | -0.06 |

All correlations p<0.01, except for the E-RS Cough & Sputum domain score and % predicted post-bronchodilator FEV_1_, p<0.05.
BDI, baseline dyspnea index; E-RS, Evaluating-Respiratory Symptoms; FEV_1_, forced expiratory volume in 1 second; SGRQ, St George’s Respiratory Questionnaire

**Figure S1** Distribution of E-RS scores at baseline, overall and by GOLD group

**
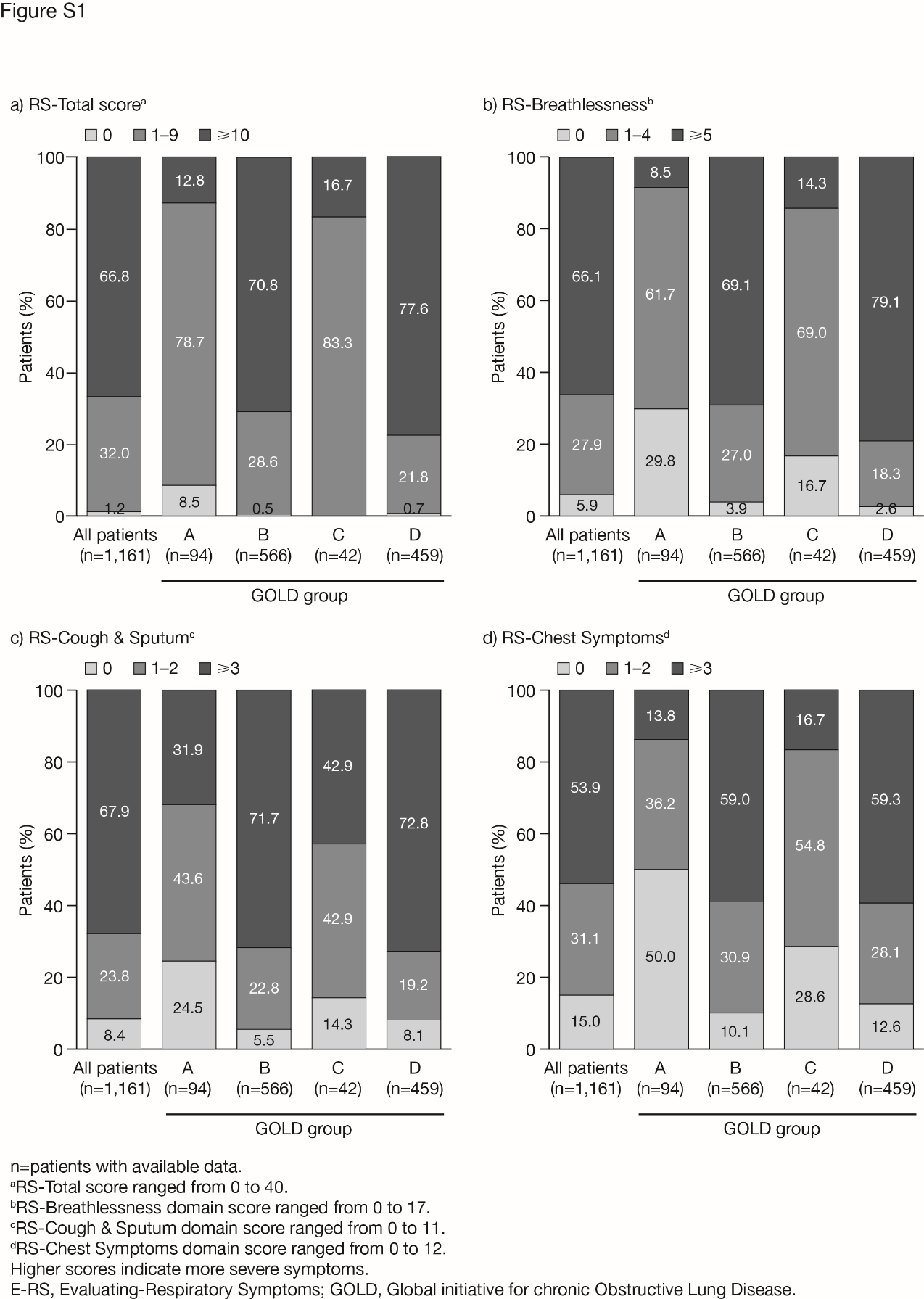
**

**Figure S2.** Relationship between patients achieving pre-defined improvements from baseline in E-RS scores and other efficacy outcomes at Week 24


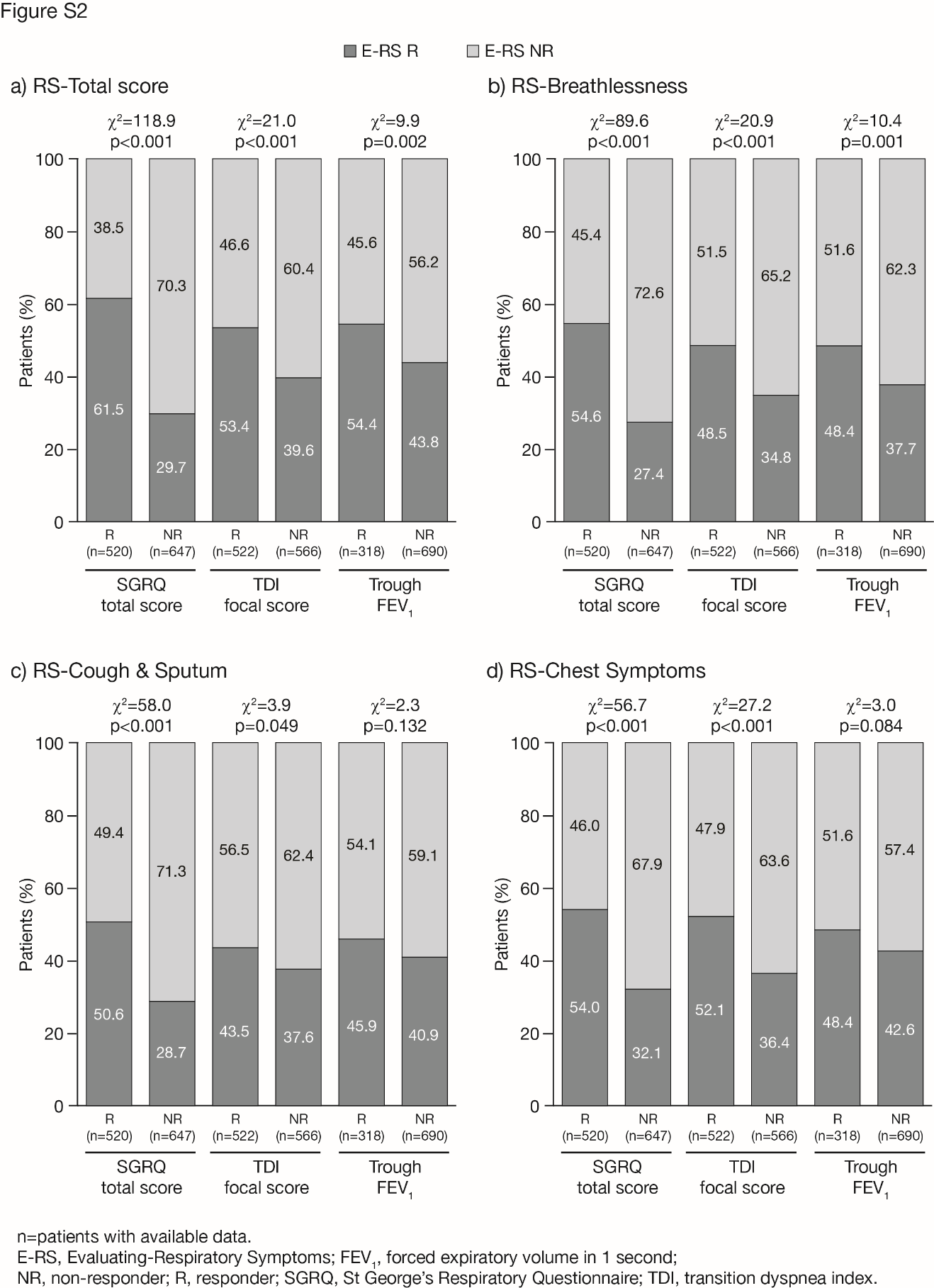

Supplement: Additional file 1: — Supplementary information. (DOCX 1244 kb) [file 12931_2016_372_MOESM1_ESM.docx]
